# Adult Cystic Intestinal Duplication of the Ileum Laparoscopically Resected after Preoperative Diagnosis with Cine Magnetic Resonance Imaging and Abdominal Ultrasound: A Case Report

**DOI:** 10.70352/scrj.cr.25-0015

**Published:** 2025-04-04

**Authors:** Takashi Takeda, Katsuki Danno, Tadafumi Fukata, Itsuko Nakamichi, Kei Yamamoto, Masaya Higashiguchi, Kozo Noguchi, Takafumi Hirao, Mitsugu Sekimoto, Yoshio Oka

**Affiliations:** 1 Department of Surgery, Minoh City Hospital, Minoh, Osaka, Japan; 2 Department of Pathology, Minoh City Hospital, Minoh, Osaka, Japan

**Keywords:** adult cystic intestinal duplication, MRI cine, abdominal ultrasound

## Abstract

**INTRODUCTION:**

Small bowel duplication in adults is an uncommon congenital anomaly that often presents with nonspecific symptoms, such as abdominal pain, vomiting, or constipation, which complicates diagnosis. Imaging techniques such as computed tomography (CT) and ultrasonography are commonly used, and cine magnetic resonance imaging (MRI) has emerged as a promising modality for diagnosing duplication cysts by capturing peristaltic movements. Surgical resection is the definitive treatment for preventing complications such as obstruction, infection, or malignant transformation.

**CASE PRESENTATION:**

A woman in her thirties visited the emergency department with persistent lower abdominal pain. Physical examination and laboratory tests, including those for tumor markers, were unremarkable. CT revealed a cystic mass near the uterus, and pelvic MRI revealed a cystic lesion that had migrated during follow-up imaging. Cine MRI showed peristaltic movements within the lesion, and abdominal ultrasonography confirmed a cystic structure with wall movements resembling intestinal peristalsis. Based on these findings, the diagnosis of a noncommunicating small bowel duplication cyst was made.

The patient underwent a laparoscopic single-port partial resection of the ileum. A cystic lesion located 75 cm proximal to the terminal ileum was excised along with a segment of the small intestine. Histopathological examination revealed a duplicated cyst lined with the small intestinal mucosa, confirming the diagnosis. The postoperative course was uneventful, and the patient was discharged 1 week postoperatively.

**CONCLUSION:**

This case highlights the utility of cine MRI and ultrasonography in the preoperative diagnosis of small bowel duplication cysts. In particular, cine MRI provides dynamic visualization of peristaltic movements within the cyst, enabling a confident diagnosis. The migration of the cyst observed on serial MRI examinations further corroborated the origin of this duplication. These findings emphasize the importance of advanced imaging modalities in the diagnosis of rare intestinal anomalies. Preoperative diagnosis of small bowel duplication cysts can be significantly enhanced by using cine MRI and ultrasonography to detect peristaltic movements. These modalities offer critical insights that aid timely surgical intervention and improve outcomes.

## Abbreviations

bSSFP
: balanced steady-state free precession
CT
: Computed tomography
MRI
: Magnetic resonance imaging
US
: ultrasonography

## INTRODUCTION

Adult small bowel duplication is a rare congenital anomaly typically diagnosed in childhood but occasionally presents in adulthood.^1–3)^ The estimated incidence of intestinal duplication ranges from 1 in 4500 to 1 in 10000 live births.^4,5)^ Although primarily diagnosed in infancy and early childhood, approximately 20% of cases present in adulthood, often with nonspecific symptoms such as abdominal pain, vomiting, or bowel obstruction.^6)^ These duplications can occur at any point along the alimentary tract, with the ileum being the most common site.^7,8)^ Morphologically, they appear as cystic or tubular structures, most often arising from the mesenteric side of the intestine, although rare cases of duplications originating from the antimesenteric side have been reported.^2)^

In adults, clinical manifestations are often nonspecific and may include symptoms such as abdominal pain, vomiting, or constipation, which can make diagnosis challenging.^2,9)^ In some cases, duplicate cysts can mimic other gastrointestinal conditions, including intussusception.^9)^ Imaging modalities such as computed tomography (CT) and ultrasonography (US) are valuable diagnostic tools, with CT being particularly effective in identifying duplication cysts and associated complications.^1,7)^ Previous studies have reported that the preoperative diagnostic accuracy for intestinal duplication ranges from 30% to 70%, depending on the imaging modality used.^6)^ While CT and US have traditionally been employed for diagnosis, recent advances in cine magnetic resonance imaging (MRI) have improved the ability to visualize peristaltic movements within the cyst wall, enhancing diagnostic confidence.

Surgical resection remains the treatment of choice for small bowel duplications not only to alleviate symptoms but also to prevent potential complications such as obstruction, infection, or malignant transformation.^7–9)^

Herein, we report a case of small bowel duplication that was successfully diagnosed preoperatively using cine MRI and abdominal US, followed by surgical resection.

## CASE PRESENTATION

A woman in her thirties presented to the emergency department with a chief complaint of persistent lower abdominal pain for several days. On physical examination, dull pain localized at the midline of the lower abdomen was observed, accompanied by mild tenderness in the same region. No signs of peritoneal irritation or palpable masses were detected and the patient was afebrile. Laboratory investigations, including complete blood count and biochemical analyses, were within normal limits. Tumor markers, including carcinoembryonic antigen and carbohydrate antigen 19-9 were also normal.

**Fig. 1:**
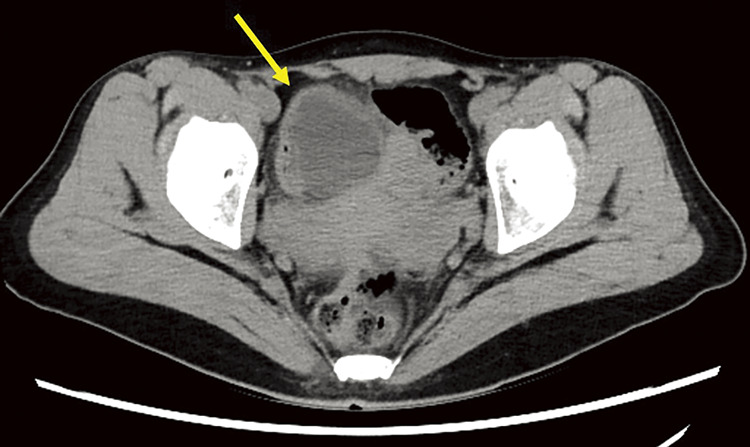
Computed tomography shows a cystic mass shadow on the right ventral side of the uterus (arrow).

**Fig. 2:**
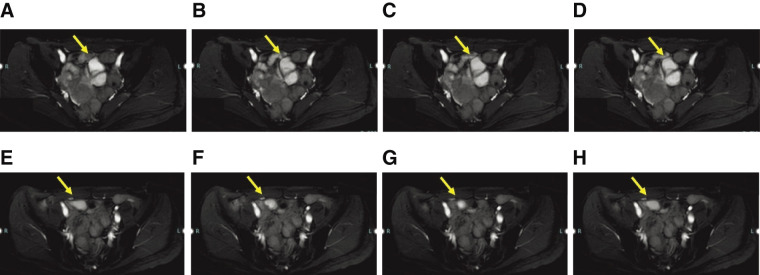
Pelvic magnetic resonance imaging showed a cystic lesion on the left side of the pelvis (arrows **A–D**). On cine imaging, the cyst appeared to be peristaltic (**A**, after 0 s; **B**, after 10 s; **C**, after 20 s; and **D**, after 30 s). Pelvic magnetic resonance imaging 1 month later showed a cyst-like structure in the right pelvis, changed from the previous one (arrows **E–H**). As before, peristalsis was observed by cine imaging (**E**, after 0 s; **F**, after 10 s; **G**, after 20 s; and **H**, after 30 s)

Plain abdominal CT revealed a cystic mass on the right anterior side of the uterus, prompting a referral to the surgical department (**Fig. 1**). The patient’s abdominal pain resolved at the time of surgical consultation. However, a plain pelvic MRI scan identified a cystic lesion on the left side of the pelvis with morphological changes, compared with the CT findings. Cine MRI sequences revealed peristaltic movements within the cyst (**Fig. 2A**–**2D**).

**Fig. 3:**
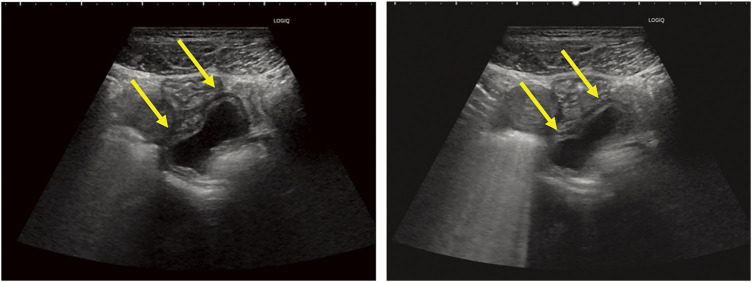
Abdominal ultrasonography showed a cystic lesion with a laminar structure and peristalsis in the upper part of the bladder.

The lower abdominal pain subsided, so she was placed under observation, but the pain recurred 1 month later, so she visited the emergency room. One month later, a follow-up plain pelvic MRI demonstrated a cystic lesion on the right side of the pelvis, indicating cyst migration. Cine MRI confirmed peristaltic movements of the cyst (**Fig. 2E**–**2D**). Abdominal US revealed a cystic lesion with a layered structure located above the bladder, accompanied by wall movements resembling intestinal peristalsis (**Fig. 3**). The interior of the cyst appeared uniformly hypoechoic without solid components. No communication between the cyst and ileum was identified. Based on these findings, the patient was diagnosed with a non-communicating small bowel duplication cyst, and surgical intervention was planned.

**Fig. 4:**
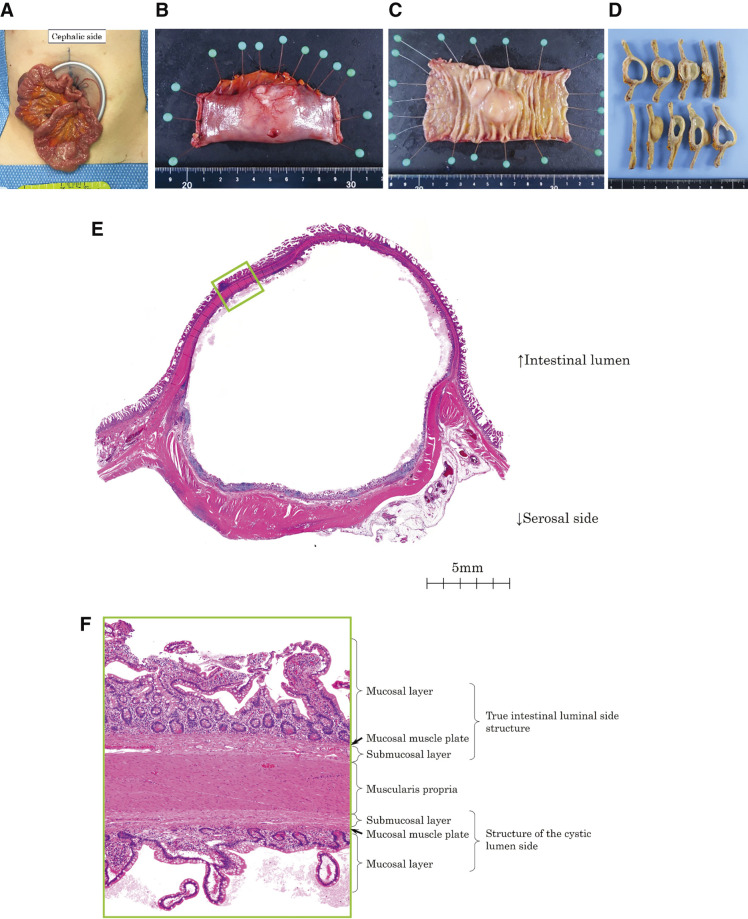
Specimen photographs and histopathology images. The cyst contained a light brown, slightly turbid fluid. There was no continuity between the cystic and ileal lumen. Histopathologically, the luminal surface of the cyst was lined with small intestinal mucosa. It had a mucosal layer, mucosal muscle plate, submucosal layer, and muscularis propria layer. The intrinsic muscular layer was interposed between the luminal side of the intestine and the cyst cavity. (**A**, intraoperative photographs; **B–D**, specimen photographs; **E–F**, histopathological images)

Laparoscopic single-port partial resection of the small intestine was performed. A 3-cm mini-laparotomy incision was made at the umbilicus to establish pneumoperitoneum. Intraoperatively, a cystic lesion was identified on the mesenteric side of the ileum approximately 75 cm proximal to the terminal ileum (**Fig. 4A**). No adhesions were observed around the cyst. The cyst and adjacent segment of the small intestine were exteriorized through the umbilical incision. A partial resection of the ileum with adequate margins from the cyst was performed (**Fig. 4B**). The procedure lasted 102 min, and the estimated blood loss was 3 mL.

Examination of the resected specimen revealed a cystic lesion on the mesenteric side of the ileum with a maximum longitudinal diameter of 35 mm (**Fig. 4C**). The cyst contained a slightly turbid, light brown fluid, and no continuity with the ileal lumen was observed (**Fig. 4D**). Histopathological analysis confirmed that the inner surface of the cyst was lined with small intestinal mucosa and included the lamina propria, submucosa, and muscularis propria (**Fig. 4E** and **4F**). These findings were consistent with the diagnosis of a small bowel duplication cyst.

The postoperative course was uneventful, and the patient was discharged 1 week postoperatively.

## DISCUSSION

Intestinal duplication in adults is a rare congenital anomaly that presents diagnostic challenges. Preoperative diagnosis is possible using imaging techniques such as US and CT. US can reveal a pathognomonic multilayered wall appearance suggestive of a digestive origin.^10,11)^ CT scans are considered to be the most sensitive diagnostic tools showing the characteristic features of intussusception and duplication.^12)^ In some cases, US may be more sensitive than CT for detecting duplications.^11)^

MRI with cine sequences has recently emerged as a promising tool for diagnosing intestinal diseases. Röttgen et al.^13)^ demonstrated that cine MRI enteroclysis provided additional clinically relevant information in 60.6% of patients, particularly in distinguishing between scarred and functional stenoses. Courtier et al.^14)^ further emphasized the value of cine MRI in confidently identifying strictures, aiding lesion detection, and assessing intestinal motility in patients with inflammatory bowel disease. These findings suggest that cine MRI techniques may offer improved diagnostic capabilities for various intestinal conditions, including adult duplication cysts.

CT is particularly useful in evaluating the anatomical location, size, and relationship of duplication cysts with adjacent structures. A characteristic feature of intestinal duplication cysts on CT is the presence of a well-defined, fluid-filled, non-enhancing cystic lesion adjacent to the gastrointestinal tract, often sharing a common muscular layer with the native bowel.^1)^ Another key diagnostic finding is the double-layered wall sign or gut signature sign, which consists of an inner hyperattenuating mucosal layer and an outer hypoattenuating smooth muscle layer, indicative of an enteric origin. This feature becomes more distinct with intravenous contrast enhancement.^8)^ However, one of the main limitations of CT is its inability to assess real-time peristalsis, which is a crucial diagnostic feature provided by cine MRI.

US is a highly effective, real-time, radiation-free imaging modality that is particularly useful in pediatric and thin adult patients for the evaluation of intestinal duplication cysts. One of the most specific sonographic findings is the gut signature sign, characterized by a double-layered wall consisting of an inner echogenic mucosal layer and an outer hypoechoic muscular layer.^11)^ This finding is considered pathognomonic for enteric duplication cysts. The cystic component of duplication cysts typically appears as an anechoic structure on US; however, internal debris may be present if hemorrhage or infection occurs.^10)^ Another significant advantage of US over CT is its ability to dynamically assess peristalsis in real time. The visualization of peristaltic movements within the cyst wall is a critical feature distinguishing duplication cysts from other cystic masses, such as mesenteric cysts or ovarian cysts.^11)^ Additionally, color Doppler US may demonstrate blood flow within the muscular wall of the cyst, further supporting the diagnosis of an intestinal duplication cyst. Although US provides valuable diagnostic information, its accuracy is highly dependent on operator expertise, and its effectiveness may be limited in obese patients or in cases where cysts are located deep within the abdominal cavity.

The preparation process for cine MRI follows the standard abdominal MRI protocols with specific modifications to optimize motion visualization. Patients are typically required to fast for at least 6 hours before the examination to reduce bowel content and minimize motion artifacts. In certain protocols, oral contrast agents such as polyethylene glycol solution may be administered to enhance the delineation of bowel loops; however, this is not always necessary for assessing duplication cysts. During the scan, the patient is positioned supine within the MRI scanner and instructed on breath-holding techniques to minimize involuntary motion. Cine MRI sequences are generally based on balanced steady-state free precession (bSSFP) techniques, such as TrueFISP (Siemens, Erlangen, Germany), FIESTA (GE Healthcare, Chicago, IL, USA), or balanced FFE (Philips, Amsterdam, Netherlands), which provide high contrast resolution between fluid-filled structures and surrounding soft tissues.^13)^

Image acquisition involves rapid, repeated sequences over a specific region of interest, typically achieving a temporal resolution of 2 to 3 frames/second, a slice thickness of 4–6 mm, and an optimized field of view for the suspected lesion site. The acquisition time for each sequence usually ranges from 30 to 60 seconds, during which continuous imaging of the target region is performed. Depending on patient tolerance, imaging can be conducted under breath-holding conditions or free breathing to capture dynamic bowel motion. This capability allows cine MRI to provide functional imaging of the intestines, offering additional diagnostic value over conventional modalities.

In the context of small bowel duplication cysts, cine MRI is particularly valuable because it allows real-time visualization of peristaltic motion within the cystic wall. This feature confirms the lesion’s digestive tract origin and helps differentiate it from other cystic masses such as mesenteric cysts or ovarian cysts. In our case, cine MRI successfully demonstrated peristaltic activity within the cyst, strongly suggesting an intestinal duplication cyst, a feature previously reported in the assessment of small bowel motility disorders.^14)^ Additionally, serial cine MRI examinations revealed cyst migration over time, further supporting the diagnosis. This dynamic functional assessment, which is not possible with static imaging modalities such as CT or conventional MRI, underscores the potential of cine MRI as a valuable adjunct in the diagnostic workup of intestinal duplication cysts.

Cine MRI has emerged as a promising imaging modality for evaluating bowel motility and detecting intestinal duplication cysts. Unlike conventional static MRI, cine MRI enables real-time visualization of peristaltic movements within the cystic wall, confirming the digestive origin of a lesion. This feature is particularly useful in differentiating duplication cysts from other cystic masses, such as mesenteric or ovarian cysts, which lack intrinsic peristalsis.^14)^ Additionally, cine MRI provides superior soft tissue contrast compared with US, allowing for precise localization and characterization of duplication cysts, including their relationship with adjacent structures.^13)^ This advantage is particularly beneficial for lesions that are deeply situated or located in anatomically challenging regions, such as the retroperitoneum. Furthermore, cine MRI allows for serial imaging to assess changes in lesion position over time. In this case, the migration of the cyst was successfully documented on sequential MRI scans, further supporting the diagnosis of an enteric duplication cyst. This ability to track dynamic changes represents a significant advantage over US and CT, which typically provide only static images. Another important benefit of cine MRI is its lack of ionizing radiation, making it a safer option, particularly for younger patients or those requiring repeated imaging.^13)^

Despite these advantages, cine MRI is not without limitations. One of the primary drawbacks is its limited accessibility, as it requires specialized imaging protocols and high-field MRI scanners, which may not be available in all medical institutions. This restricts its widespread clinical application, particularly in resource-limited settings. Additionally, cine MRI is associated with higher costs compared with conventional imaging techniques, such as CT and US, which may further limit its routine use.^13)^ Another disadvantage is the longer examination time and the need for patient cooperation. MRI requires the patient to remain still for an extended period, which can be uncomfortable, especially for individuals experiencing significant abdominal pain. By contrast, US can be performed rapidly at the bedside with minimal patient burden.^14)^ Moreover, cine MRI is susceptible to motion artifacts caused by respiratory movements and bowel peristalsis outside the target area. While breath-holding techniques can mitigate these effects, they may not be feasible for all patients, particularly those with respiratory compromise or severe discomfort. MRI examinations, particularly cine sequences, require longer scanning times and specialized radiological expertise, contributing to increased healthcare expenditures.

When compared with US, cine MRI offers superior tissue contrast and functional assessment, particularly for deep-seated lesions. However, US remains a highly valuable and practical alternative due to its real-time capability, cost-effectiveness, and accessibility. US can directly visualize intestinal peristalsis using Doppler or grayscale imaging, making it an effective alternative for assessing duplication cysts.^11)^ Additionally, US is widely available, portable, and does not require extensive patient preparation, making it a more practical choice for initial evaluations.

Although often discovered incidentally, early diagnosis through imaging is crucial for the timely management and prevention of complications.^12)^ However, in some cases, a definitive diagnosis may require surgical confirmation and histopathological analysis.^11,12,15)^

Compared with previous reports, our case highlights the clinical utility of cine MRI in the preoperative diagnosis of adult intestinal duplication cysts. Cine MRI enabled the visualization of peristaltic movements within the cyst, a feature indicative of its digestive origin. This observation is consistent with the findings of Guibaud et al., who reported the diagnostic value of wall movements in identifying duplications.^11)^ Although CT and US have traditionally been the preferred modalities for diagnosing intestinal duplications, our findings suggest that cine MRI offers additional advantages, particularly in cases where dynamic wall motion provides critical diagnostic information.

Moreover, this case underscores the significance of cyst migration observed on serial MRI examinations. Previous studies have not extensively addressed the role of serial imaging in identifying such features, emphasizing the novelty and diagnostic value demonstrated in this case.

Upon reviewing the existing literature, the application of cine MRI in diagnosing intestinal duplication cysts is exceedingly rare. While cine MRI has been utilized to assess small bowel motility in various gastrointestinal disorders, including inflammatory bowel disease and intestinal strictures, its specific use in identifying peristaltic activity within duplication cysts has not been well-documented. This case represents a novel application of cine MRI, providing dynamic visualization of peristalsis within the cyst wall, thereby confirming its enteric origin. To our knowledge, this is one of the first reported instances where cine MRI has been employed preoperatively to diagnose an intestinal duplication cyst, distinguishing it from other cystic abdominal lesions.

In this case, the surgical intervention was both diagnostic and therapeutic. The absence of adhesions and histopathological confirmation of the diagnosis aligns with the existing literature on noncommunicating duplication cysts. This case illustrates the importance of integrating advanced imaging techniques and minimally invasive surgical approaches to optimize the outcomes of patients with intestinal duplication cysts. In this case, a laparoscopic single-port partial resection of the small intestine was performed, which contributed to the patient’s uneventful postoperative course and rapid recovery. The minimally invasive approach allowed for precise dissection and complete resection of the duplication cyst while minimizing surgical trauma.

## CONCLUSION

Here, we report the case of a small bowel duplication cyst that was successfully diagnosed preoperatively using imaging techniques. When a cystic mass suggestive of intestinal duplication is identified in the abdominal cavity, cine MRI and abdominal US, which detect peristaltic movements, can serve as valuable tools for aiding preoperative diagnosis. These imaging modalities may enhance diagnostic accuracy and inform surgical planning in cases of suspected small bowel duplication.

## ACKNOWLEDGMENTS

The authors thank Editage (www.editage.jp) for English language editing.

## DECLARATIONS

### Funding

This study did not receive any funding or grants.

### Authors’ contributions

The study conception and design were performed by TT and KD.

Data acquisition was performed by TT, TF, and IN.

TT, KY, MH, and KN drafted the manuscript.

The manuscript was critically revised by TH, MS, and YO.

All authors have read and approved the manuscript.

### Availability of data and materials

All data generated or analyzed during this study are included in this published article.

### Ethics approval and consent to participate

Informed consent was obtained from the patient to be included in the study. This work did not require ethical considerations or approval. All procedures were performed in accordance with the ethical standards of the responsible committee on human experimentation (institutional and national) and the Helsinki Declaration of 1975, as revised in 2008 (5).

### Consent for publication

Informed consent for publication of this case report was obtained from the patient.

### Competing interests

The authors declare that they have no competing interests.
